# A novel membrane stress response that blocks chromosomal replication by targeting the DnaA initiator via the ClpP protease

**DOI:** 10.1128/jb.00151-25

**Published:** 2025-06-24

**Authors:** Alabi Gbolahan, Tong Li, Rishit Saxena, Karen Wolcott, Aamna Sohail, Ishika Ahmed, Dhruba K. Chattoraj, Elliott Crooke, Rahul Saxena

**Affiliations:** 1 Department of Biochemistry and Molecular & Cellular Biology, Georgetown University Medical Center 12231, Washington, DC, USA; 2 Basic Research Laboratory, Center for Cancer Research, National Institutes of Health, National Cancer Institute 2511, Bethesda, Maryland, USA; 3 Lombardi Comprehensive Cancer Center, Georgetown University Medical Center 12231, Washington, DC, USA; Queen Mary University of London, London, United Kingdom

**Keywords:** DNA replication, membrane stress, ClpP protease, stress response, (p)ppGpp, Lon protease

## Abstract

In *Escherichia coli*, membrane stress due to interrupted lipoprotein (Lpp) maturation impairs DNA replication and arrests cell growth. How Lpp maturation and DNA replication are connected remains unclear. We demonstrate that upon membrane stress, the Rcs stress-response pathway is activated, and the replication initiator DnaA is lost, which explains the replication block. However, Lon protease, a key regulator of the Rcs pathway, is not required for the DnaA loss. We further ruled out the involvement of (p)ppGpp, one of the major mediators of stress responses in bacteria. On the other hand, upon deletion of the ClpP protease gene, DnaA was stable, replication was not inhibited, and there was no cell-growth arrest. In wild-type cells, overexpression of DnaA was lethal even without the membrane stress apparently from hyperinitiation. In ∆*crp* cells, hyperinitiation was restrained, and overexpression of DnaA was able to overcome the growth arrest. *∆fis* cells, which were earlier found resistant to the membrane stress, showed DnaA stability and normal replication upon stress-inducing treatments. We conclude that DnaA loss suffices to explain the growth arrest upon the membrane stress. The stress-response pathway described here appears novel because of its independence from Lon and (p)ppGpp, which have been implicated in other stress responses that block DNA replication.

**IMPORTANCE:**

The observation that DNA replication stress can block cell division in *E. coli* (SOS response) introduced the concept of checkpoint control in the cell cycle. Here, we describe a novel checkpoint control that functions in the opposite direction: membrane stress causing replication block. We show how the accumulation of precursor lipoprotein (pLpp) could block replication. The pLpp accumulation causes a response culminating in activating the ClpP protease that blocks replication by targeting the initiator DnaA. DnaA being vital and highly conserved, a detailed understanding of the response pathway is likely to open new avenues to treat bacterial infection.

## INTRODUCTION

**Fig 1:**
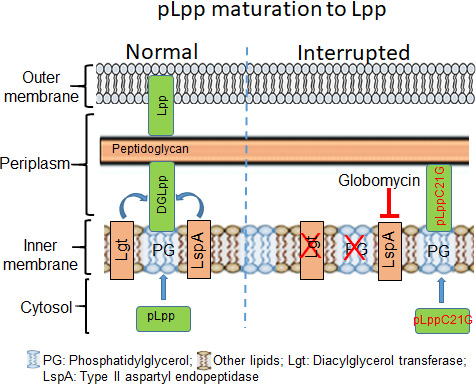
Schematic representation of the Lpp maturation pathway and how it is interrupted at different steps. Lpp is synthesized as pLpp, interacts with PG, and gets modified by Lgt to DGLpp. DGLpp is processed by LspA and others, and mature Lpp is incorporated into the outer membrane. The maturation is interrupted (indicated in red) either by mutating Lgt, depleting PG, inactivating LspA by Globomycin, or expressing a processing-defective *lpp*(C21G) mutant. The interrupted products accumulate in the inner membrane, causing membrane stress.

In Gram-negative bacteria such as *Escherichia coli*, the cell envelope consists of two membranes, one outer (OM) and one inner (IM) with a periplasmic space in between wherein lies the peptidoglycan layer, the cell wall (Fig. 1). The OM has lipid-attached proteins (lipoproteins) that are an evolutionarily conserved family of acylated proteins. These serve several functions, including biogenesis of membranes and sensing of stress from external or internal stimuli (1, 2). In *E. coli*, the most abundant lipoprotein, Lpp (~10^6^ molecules per cell), provides structural support to the OM by crosslinking it to the peptidoglycan layer (3, 4). The synthesis of nascent Lpp (product of the *lpp* gene) occurs in the cytosol as prelipoprotein (pLpp), which is located in the IM (1–4) (Fig. 1). There, pLpp is modified at its Cys21 residue by the addition of diacylglycerol (DG) from phosphatidylglycerol (PG), the major anionic phospholipid of the IM (5–8). This lipidation of pLpp is catalyzed by diacylglycerol transferase, Lgt (9). The resultant DGLpp is cleaved by the IM-bound type II aspartyl endopeptidase, LspA (10). The cleaved product is processed further to produce the mature Lpp that translocates to the OM (1–8). Although Lpp provides structural support to the OM by crosslinking it to the peptidoglycan layer, Δ*lpp* mutants are viable (11). Nevertheless, interrupting the processing of pLpp at various steps of its maturation results in the accumulation of intermediates in the IM, and *E. coli* becomes non-viable (9–14) (Fig. 1).

An early indication of unprocessed pLpp accumulation causing lethality in *E. coli* came from the repression of the PG synthase A (*pgsA*) gene expression (“*pgsA*-null”), which would prevent the DG modification step of pLpp (12, 13). Deletion of the *lpp* gene of *pgsA*-null cells allowed their growth, indicating that the growth arrest due to reduction in PG levels could be associated with defective Lpp biogenesis (15, 16). The Lpp biogenesis in wild-type *E. coli* (*pgsA^+^*) could also be interrupted by deleting the *lgt* gene (9) or overexpressing mutant pLpp(C21G), either of which would interfere with the DG modification step (11, 14), or by blocking LspA enzyme activity, which cleaves the IM-bound DGLpp, using a lipopeptide inhibitor, globomycin (17, 18) (Fig. 1). In sum, membrane stress from interrupted lipoprotein biogenesis in any of the steps of the pLpp maturation pathway can cause growth arrest.

The growth-arrested *pgsA-*null cells also have decreased frequency of replication initiation (19, 20). Here, we have investigated how blockage of Lpp maturation could block chromosomal DNA replication.

Biochemical studies have shown that membrane lipids containing anionic head groups, like PG and CL (cardiolipin), can rejuvenate inactive initiator DnaA-ADP to active initiator DnaA-ATP (21). Imaging studies have indicated that DnaA is associated peripherally to the membrane (22–24). In fact, DnaA has a stretch of amino acid residues in its ATPase (AAA^+^) domain that can bind membrane (25, 26). That the membrane state might be involved in DnaA function is suggested by the finding that the overexpression of DnaA(L366K), where the changed residue is in the membrane-binding stretch of the AAA+ domain, suppresses growth arrest due to defective Lpp maturation (as in *pgsA-*null cells or wild-type *[pgsA*^+^] cells overexpressing pLpp(C21G)) (14, 25–27). Similarly, we have recently shown that overexpression of DnaA with small deletions in its linker domain, a domain that also has membrane-binding capacity, can overcome the growth arrest (28). These results suggest that the growth arrest from defective Lpp biogenesis could be mediated by inactivating DnaA.

Transient faults in several cellular processes often induce responses (stress responses), which buy time for cells to adapt to stressful conditions. A well-studied stress response is mediated via the Rcs (Regulator of capsule synthesis) pathway, which is activated primarily due to defects in lipopolysaccharide (LPS) synthesis (29–31). Three positive regulators, RcsA, RcsB, and RcsF, and two negative regulators, RcsC and Lon (29, 30), control the Rcs stress response. The Lon protease degrades RcsA to downregulate capsular polysaccharide synthesis under normal growth (30). *∆lon* cells are mucoid due to increased *cps* gene expression apparently from unchecked RcsA levels in the absence of Lon (31, 32).

In *pgsA*-null cells, induction of the CpxAR system is also known (33). The system monitors the trafficking of OM-targeted lipoproteins and responds to their mislocalization in IM via activation of the DegP protease (34). The extracytoplasmic stress is relieved by activation of ClpP (caseinolytic protease P). ClpP functions with ATPases, ClpA, or ClpX by forming ClpAP or ClpXP heterodimers, which can degrade damaged, misfolded, and regulatory proteins and thus help maintain protein quality control (35).

Various stressful conditions, such as nutrient limitation, oxygen free-radical production, fatty acid limitation, and antibiotic treatment (36–38), induce the synthesis of nucleotide-based second messengers. Such messengers include guanosine tetra- and pentaphosphate, collectively known as (p)ppGpp, the hallmark of stringent response in bacteria (39). (p)ppGpp inhibits the activity of PPX (exopolyphosphatase), which hydrolyzes polyphosphate (PolyP) (39). Accumulation of PolyP may activate the Lon protease, which can degrade DnaA (40, 41). (p)ppGpp can also inhibit initiation of DNA replication in other ways, for example, by altering DNA topology of the replication origin region (42).

In this study, the growth arrest of *E. coli* under membrane stress due to interrupted Lpp-maturation appears to be due to ClpP-mediated proteolysis of DnaA and thereby blocking of new rounds of replication initiation. Conditions were found where the growth arrest upon the membrane stress could be overcome: DnaA overexpression in *∆crp* cells and without overexpression in *∆fis* cells. Although the mechanistic details remain to be studied, DnaA was stable in these conditions. The results demonstrate that DnaA loss is responsible for cell growth arrest upon induction of the membrane stress.

## RESULTS

### DnaA is lost in cells growth-arrested due to interrupted lipoprotein biogenesis

In *E. coli*, the requirement of PG is evident both for processing pLpp (8–11) and for chromosomal replication (19, 20). We showed that, as opposed to *lpp*(WT), expression of a mutant gene *lpp*(C21G), whose product cannot be processed and that accumulates in the IM, causes growth arrest of wild-type *E. coli* cells (14). The growth arrest could be overcome by overexpression of not the WT DnaA but a mutant DnaA, where the change is in one of the membrane-interacting interfaces of the initiator (14, 27, 28). These studies implied that the membrane stress response causing the growth arrest could be mediated through DnaA, but in what way remained an open question.

**Fig 2:**
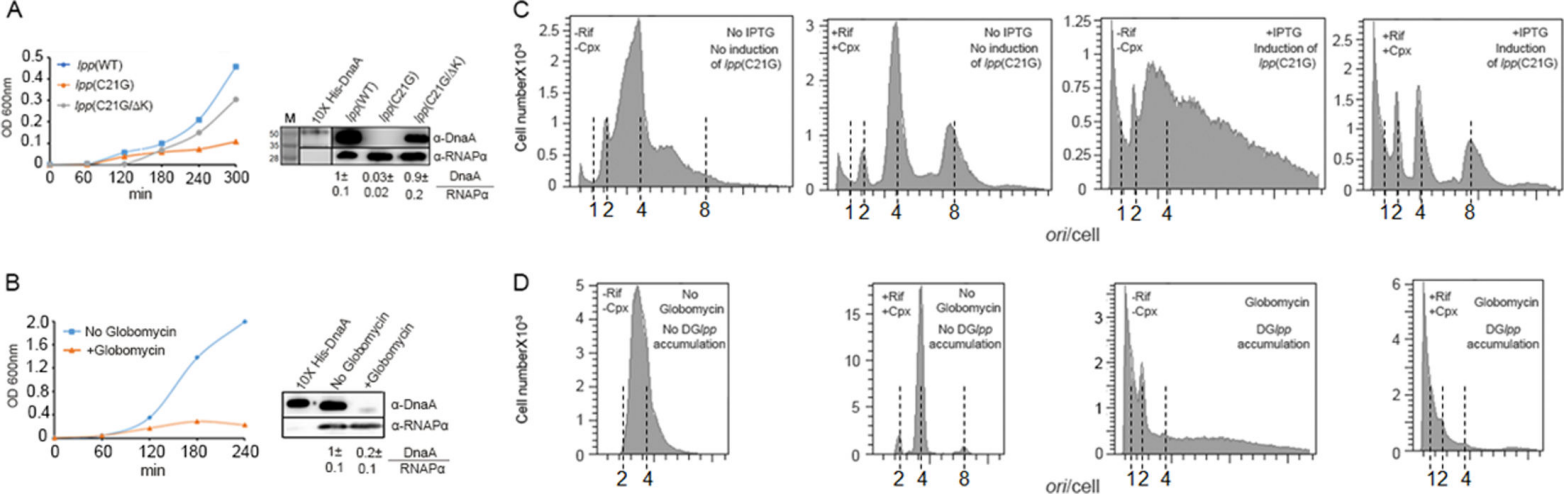
Membrane stress upon interrupting Lpp maturation results in loss of DnaA initiator and block of replication initiation. (**A**) Growth of wild-type (*dnaA*^+^) cells expressing *lpp*(WT), *lpp*(C21G), and *lpp*(C21G∆K) genes (*left panel*). Immunoblotting (*right panel*) of DnaA (52 kDa) and α-subunit of RNA polymerase (28 kDa used as loading control) from cells used in the left panel. M indicates the protein size marker lane. Purified 10× His-tagged DnaA was used as the DnaA size marker. Numbers indicate the relative ratios of DnaA to RNAPα proteins. The DnaA/RNAPα ratios are normalized w.r.t. the *lpp*(WT) value of 1. (**B**) Growth of wild-type cells in the absence and presence of globomycin (*left panel*). Immunoblotting of DnaA and RNAPα from such cells (*right panel*). Other details are the same as in (**A**). (**C*,* D**) Histogram of DNA content per cell before (-Rif/-Cpx) and after (+Rif/+Cpx) replication run-out analyzed by flow cytometry. Numbers in the abscissa indicate chromosomal origins per cell in exponentially growing cells or at the time of drug addition. 100,000 cells were counted in all flow cytometry experiments. Positions of *ori*/cell were estimated as described in Materials and Methods.

To address this, we used plasmids to overproduce Lpp(WT) and pLpp(C21G) proteins from the inducible P*lac* promoter by including the inducer IPTG in the growth medium (14). In addition to Lpp(WT), we used another negative control, the pLpp(C21G/∆K) protein. Normally, pLpp covalently crosslinks to the peptidoglycan layer using the carboxy-terminal lysine, deletion of which keeps the mutant protein (pLpp(C21G/∆K)) cytosolic and thereby does not cause growth arrest (43). Upon the addition of 50 µM IPTG, cells expressing pLpp(C21G) grew up to an OD_600_ of 0.10, whereas the cells expressing the negative controls Lpp(WT) and pLpp(C21G/∆K) reached a higher OD_600_ of 0.3–0.5 in the same time interval (Fig. 2A, *left panel*). The generation time also increased ~1.4-fold when cells were expressing pLpp(C21G) as opposed to Lpp(WT) or pLpp(C21GΔK) (Table S1). Immunoblotting data using polyclonal α-DnaA-antiserum suggest that DnaA protein is lost in cells expressing pLpp(C21G) and not in cells expressing Lpp(WT) or pLpp(C21G/∆K) (Fig. 2A, *right panel*). When pLpp maturation was blocked using globomycin, the generation time also increased compared to that of cells with no drugs (Fig. 2B, *left panel* and Table S2). We also confirmed that the globomycin-induced toxicity requires Lpp since *lpp*^+^ cells showed increased toxicity against globomycin as compared to *Δlpp* cells, as was suggested in a previous study (18,Fig. S1A). The immunoblotting data showed that, as in the wild-type (*pgsA^+^ lpp^+^* ) cells expressing *lpp*(C21G), DnaA was lost when the wild-type cells were treated with globomycin (Fig. 2B, *right panel*). The growth arrest can thus be attributed to the DnaA loss. In the remainder of this study, membrane stress will be studied mainly in wild-type (*pgsA^+^ lpp^+^*) cells either by expressing plasmid-borne *lpp*(C21G) gene or by treatment with globomycin.

The loss of DnaA under the membrane stress is expected to block replication initiation at the chromosomal *oriC*. This was tested by flow cytometry. When wild-type cells contained the stressor plasmid (i.e., the one carrying the stressor gene *lpp*(C21G)), but the stressor expression was not induced, discrete peaks were seen in the DNA histogram when a single bell-shaped curve was expected (44, Fig. 2C, *first panel*). The appearance of discrete peaks suggests some replication initiation blockage, which could be from leaky expression of the stressor. Indeed, our wild-type cell without carrying the stressor plasmid showed the expected distribution with a single broad peak (Fig. 2D, *first panel*). The leakiness of stressor expression was tested by replacing the stressor gene with the chloramphenicol acetyltransferase gene (*cat*) under the same P*lac* promoter. Cells transformed with the resultant plasmid, which also carried the *bla* gene and selected for Amp^R^, showed resistance to chloramphenicol when tested without inducing *cat* expression (Fig. S1B).

Upon replication run-out using rifampicin and cephalexin, the DNA peaks became sharper, particularly in wild-type cells without plasmids, representing cells with full 2, 4, and 8 chromosomes, indicating synchronous initiation (Fig. 2D, *second panel*). Compared to wild-type cells, replication initiation was more asynchronous in the stressor-carrying cells, a conspicuous feature being the appearance of cells with one or, most likely, less than one chromosome equivalent of DNA (Fig. 2C, *first two panels*). The ambiguity is because the expected position of the one-chromosome peak (deduced from the reference peaks of cells with 2 and 4 chromosomes) did not match the observed peak. If the stressor is causing DnaA loss, this ought to prevent new initiation, and there should be cells with one chromosome, even without requiring rifampicin to block new initiation. The cells with DNA content apparently less than one chromosome, if true, indicate some DNA breakdown upon induction of the stress. The “one-chromosome” peak was more prominent upon induction of the stressor (Fig. 2C, *last two panels*). Stress induction also reduced the four-chromosome peak in favor of the two-chromosome peak, indicating blockage of new initiation. (Fig. 2C, the *second* and the *last panels*).

Results were similar when, instead of the stressor *lpp*(C21G), globomycin was used to block pLpp maturation (Fig. 2D, *last two panels*). Globomycin was more effective in blocking replication (Fig. 2C vs. D). This could be because the drug action was more immediate than that which could be achieved in an inducible system, where the inhibitor needed to be synthesized and accumulated. Blocking LspA could also be more effective in inducing the membrane stress (Fig. 1). These results indicate that the interrupted trafficking of a nonessential lipoprotein, Lpp, causes DnaA loss that blocks the vital event of replication initiation.

**Fig 3:**
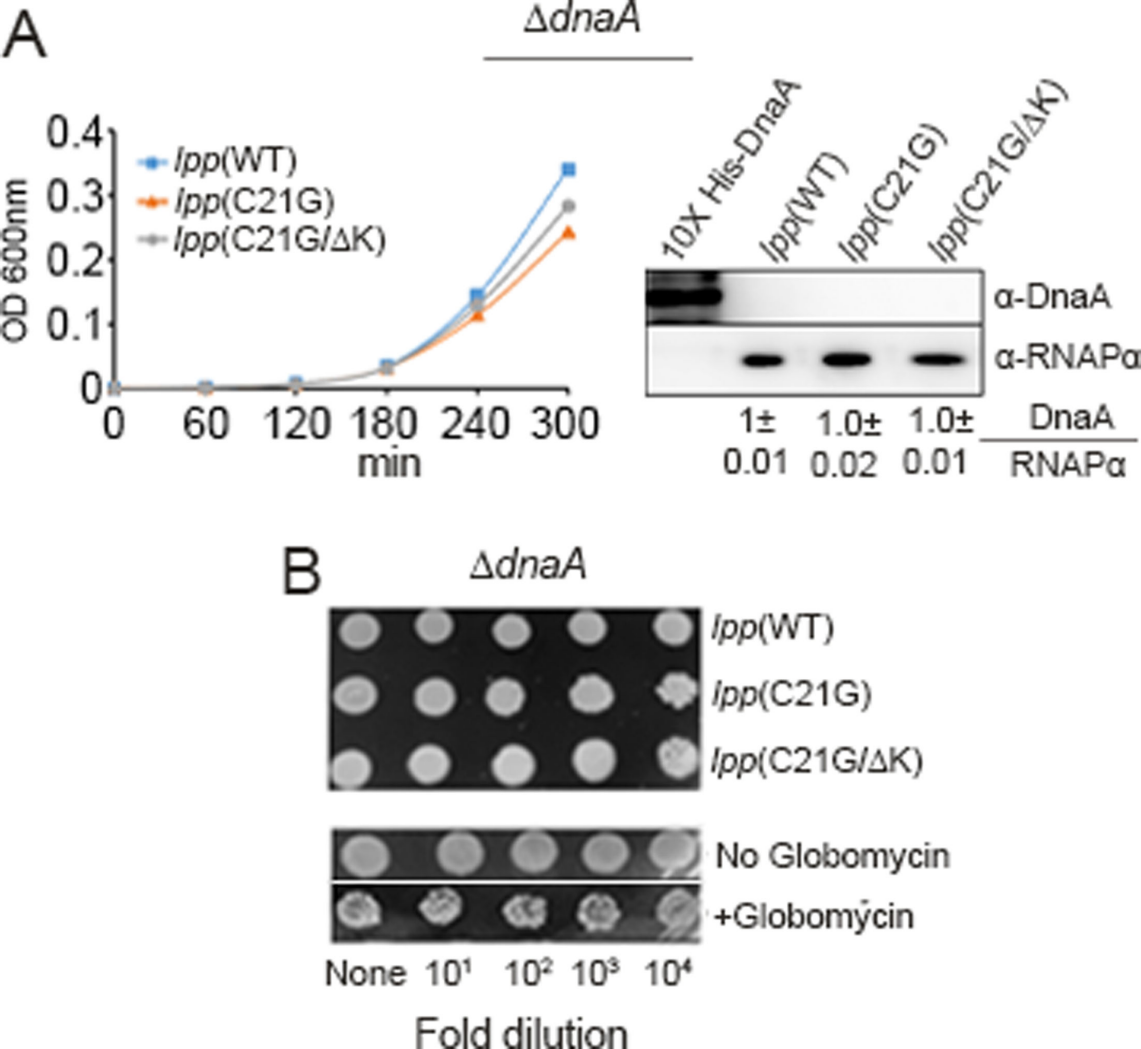
Membrane stress upon interrupting Lpp maturation is DnaA-dependent. (**A**) Growth of ∆*dnaA* cells expressing *lpp*(WT), *lpp*(C21G), and *lpp*(C21G∆K) genes (*left panel*) and immunoblotting of DnaA and RNAPα from such cells (*right panel*). (**B**) Spotting assay to test viability of ∆*dnaA* cells expressing *lpp*(WT), *lpp*(C21G), or *lpp*(C21G∆K) genes or when the cells were exposed or not to globomycin. Note that the lower panel is a composite of pictures from two different plates (±globomycin).

The requirement for the *oriC* region and the *dnaA* gene, although normally essential for replication initiation, can be bypassed by other means (45–47). For example, *E. coli* ∆*dnaA* cells can be made viable by integrating into the chromosome a miniR1 plasmid (pKN500), where the replication initiates from the plasmid origin independently of DnaA (47). We asked whether, in these plasmid-integrated ∆*dnaA* cells, the growth arrest in response to membrane stress could be bypassed, if the growth arrest were due to blockage of DnaA-mediated replication initiation. We transformed the ∆*dnaA* cells with plasmids expressing Lpp or its mutants. As expected, overexpression of *lpp*(C21G) did not affect the growth of ∆*dnaA* cells, unlike the situation in wild-type cells (Fig. 3A, *left panel* and 3B, *top panel*). We confirmed by immunoblotting that no band corresponding to DnaA protein is present in any of the transformants of ∆*dnaA* cells (Fig. 3A, *right panel*). Similarly*,* ∆*dnaA* cells were resistant to globomycin, as opposed to wild-type cells (Fig. 3B, bottom *panel*). In addition, in ∆*dnaA* cells, no significant differences in growth rate were found when the WT or mutant Lpp proteins were expressed (Table S1). We conclude that the interrupted trafficking of a nonessential lipoprotein, Lpp, perturbs specifically the DnaA-dependent growth arrest.

### Membrane stress activates the Rcs stress response

Stresses to cells due to internal dysfunction or external insults can induce responses (stress responses), which buy time to repair damages from the stress and help cells return to normalcy. One of the best-studied membrane stress-response pathways is the Rcs (regulator of capsule synthesis) pathway. This pathway involves transcriptional activation of a complex network of capsular polysaccharide (*cps*) genes controlling the production of colonic acid, which makes cells mucoid (29, 30). The protease Lon serves as the negative regulator of colonic acid production. Lon degrades RcsA, one of the subunits of the transcriptional activator RcsA-RcsB heterodimer and thus inhibits expression of *cps* genes and prevents cells from becoming mucoid. *∆lon* cells are, therefore, mucoid (31).

**Fig 4:**
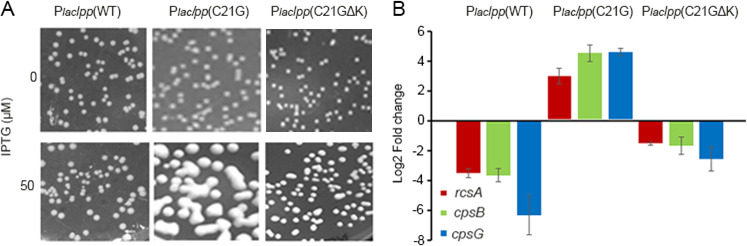
Membrane stress upon interrupting Lpp maturation induces the Rcs stress response. Plasmids carrying *lpp*(WT), *lpp*(C21G), or *lpp*(C21G∆K) genes placed under the P*lac* promoter were used to transform wild-type (*dnaA^+^*) cells. (**A**) Growth phenotype of cells expressing WT and mutant Lpp genes in the presence of 0 or 50 µM IPTG. (**B**) qRT-PCR analysis of *rcsA*, *cpsB,* and *cpsG* mRNA in cells expressing *lpp*(WT), *lpp*(C21G), or *lpp*(C21G∆K) genes. Data were normalized to the ∆Cq value of a reference gene *rrsA*.

When spread on agar plates containing IPTG (50 µM), we found that stressed cells, that is, those expressing the plasmid-borne *lpp*(C21G) gene, exhibit the mucoidy phenotype (Fig. 4A, *middle panel*). Mucoidy was not seen in cells expressing the negative controls, *lpp*(WT) and the *lpp*(C21G/ΔK) genes (Fig. 4A, *left* and *right panels*, respectively), suggesting that mucoidy is specific to cells expressing pLpp(C21G). The activation of the Rcs stress response is known in *pgsA*-null cells, where pLpp maturation is expected to be blocked, causing it to accumulate in the membrane and stress it (32). To test whether the Rcs pathway is on in mucoid cells expressing pLpp(C21G), we isolated total RNA from these cells (collected from plates) and performed qRT-PCR analysis. We found that mucoid cells expressing *lpp*(C21G) have increased levels of mRNA belonging to *rcsA*, *cpsB,* and *cpsG* genes (Fig. 4B). These results confirm activation of the Rcs pathway when cells are under stress due to *lpp*(C21G) expression.

### Lon protease is not required for DnaA loss when cells are under membrane stress

Our results so far indicate that the membrane stress upon interrupted lipoprotein biogenesis activates the Rcs stress-response pathway and causes DnaA loss. However, the cause of the loss remains unknown. *E. coli* under stressful conditions, such as nutrient depletion, antibiotic treatment, and defective oxidative phosphorylation, often accumulates (p)ppGpp molecules, a hallmark of stringent response (36–38). (p)ppGpp activates the Lon protease in *Caulobacter crescentus* (48, 49) and *E. coli* (40, 41) that can degrade DnaA, blocking new rounds of replication. These results prompted us to investigate whether blocking stringent response or deleting the *lon* gene could allow cells to tolerate membrane stress caused by *lpp*(C21G) expression.

**Fig 5:**
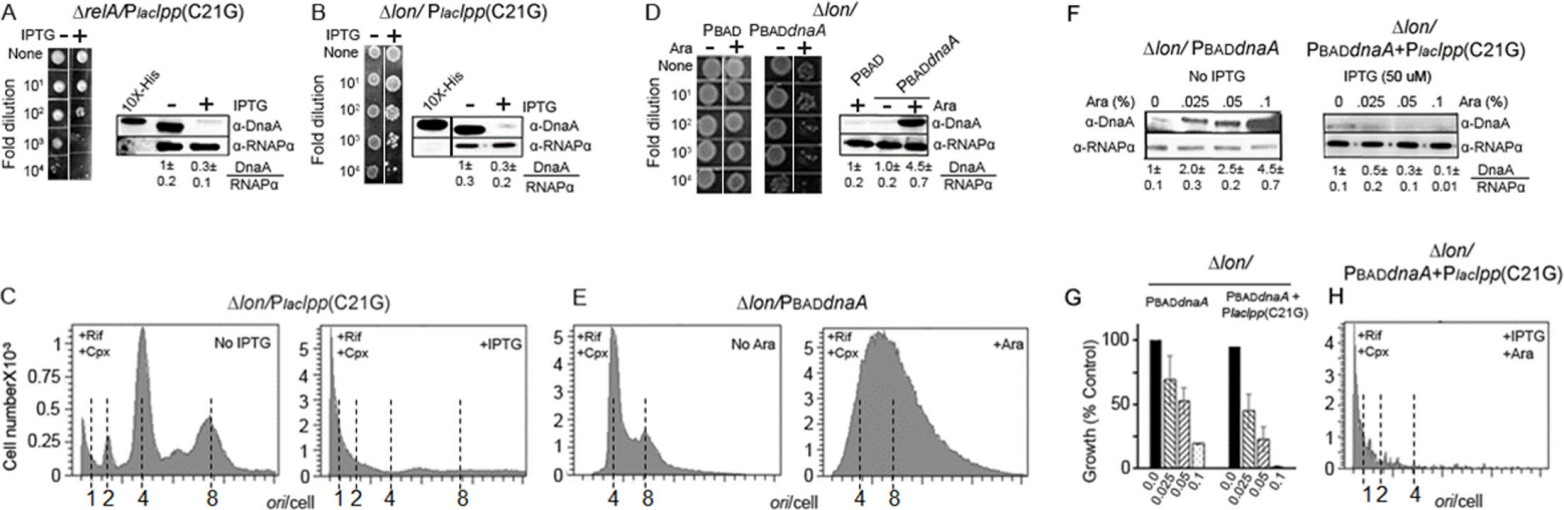
Depletion of DnaA upon membrane stress is independent of (p)ppGpp alarmone and Lon protease and is not overcome by overexpressing DnaA. (**A, B***) E. coli* Δ*relA* (the gene required for (p)ppGpp synthesis) or Δ*lon* cells, transformed with a plasmid carrying the stressor gene P*laclpp*(C21G), were grown to an OD_600_ of 0.10, and the cultures were serially diluted. 2.5 µL from each dilution was spotted on M9-Agar plates without and with 50 µM IPTG to express the stressor gene (*left panels*). Note that the results from two different plates (±IPTG) are presented as a single composite picture. Growth should be compared vertically within a column rather than between columns. Immunoblotting to measure DnaA content of ∆*relA* and ∆*lon* cells expressing the stressor (*right panels*). Other details are the same as in Fig. 2A. (**C**) Replication run-out profiles of ∆*lon* cells carrying the stressor plasmid in the absence (*left panel*) and presence (*right panel*) of IPTG, as in (**B**). (**D**) Growth of *∆lon* cells carrying plasmids containing PBAD or PBAD*dnaA*. Their growth in the absence (*left panel*) or presence (*middle panel*) of arabinose by spotting assay and DnaA content by immunoblotting (*right panel*). (**E**) Origin content of the same cells as in (**D**), as measured by flow cytometry. (**F**) DnaA is depleted upon the membrane stress, even when over-expressed. Δ*lon* cells carrying the P_BAD_*dnaA* plasmid were grown to an OD_600_ of 0.10 and plated on M9-Agar plates containing appropriate antibiotics and arabinose at various concentrations (0%, 0.025%, 0.05%, and 0.1%). Single colonies from such plates were inoculated in liquid media with identical supplements, and DnaA content of the cells was determined by immunoblotting (*left panel*). The ∆*lon/*PBAD*dnaA* cells were further transformed with the stressor plasmid with the gene P*laclpp*(C21G), and transformants were selected with appropriate antibiotics but without adding any inducer. The transformants were used to inoculate liquid media as before, but in the absence or presence of 50 µM IPTG before the cultures were probed for DnaA (*right panel*). Other details are the same as in (Fig. 2A). (**G**) Cell viability of Δ*lon* cells expressing DnaA or co-expressing *dnaA* and *lpp*(C21G) genes. (**H**) Replication run-out profile of Δ*lon* cells co-expressing *dnaA* and *lpp*(C21G) genes, as in (**G**).

To test this, we used *E. coli* ∆*relA* and ∆*lon* cells. In ∆*relA* cells, the stringent response is not induced because RelA is required for (p)ppGpp synthesis (50, 51). We transformed ∆*relA* and ∆*lon* cells with the stressor plasmid. However, similar to wild-type cells, the growth for both ∆*relA* and ∆*lon* cells was inhibited when the inducer (50 µM IPTG) was included in the medium (Fig. 5A and B, *left panels*, respectively). Immunoblotting data showed that both ∆*relA* and ∆*lon* cells expressing *lpp*(C21G) have lost DnaA (Fig. 5A and B, *right panels*, respectively). Flow cytometry profiles, tested in the case of ∆*lon* cells, indicated a block of chromosomal replication initiation, as would be expected if DnaA were lost (Fig. 5C *left* vs. *right panels*). Together, these results indicate that (p)ppGpp and Lon activities are not essential for the DnaA loss and blocking of replication due to interrupted lipoprotein maturation.

It has been recently reported that inhibition of new rounds of DNA replication due to stringent response is not solely due to the lowering of DnaA content, as overproduction of DnaA from plasmids does not remove the replication block (52). These results led us to examine whether in *∆lon* cells, supplying extra DnaA from a plasmid source could compensate for the DnaA loss due to the membrane stress. For this, ∆*lon* cells were transformed with plasmids carrying PBAD or PBAD*dnaA*. As expected, when the PBAD promoter was not induced, the growth of bacteria containing PBAD and PBAD*dnaA* plasmids remained unaffected (Fig. 5D, Ara(−) *columns*). However, when the PBAD promoter was induced, growth of *∆lon* cells was inhibited when they carried the PBAD*dnaA* plasmid (Fig. 5D, Ara(+) columns). Immunoblotting confirmed the overproduction of the WT DnaA protein when the inducer was present (Fig. 5D, *right panel*). In flow-cytometric analysis, the replication initiation appeared excessive in ∆*lon* cells overproducing DnaA(WT) (Fig. 5E, *left* vs. *right panels*). These results raised the possibility that the lethality due to DnaA(WT) overproduction in wild-type (14, and Fig. S1C) and *∆lon* cells (Fig. 5D) could be a consequence of hyper-replication initiation.

An earlier report also suggested that overproduction of DnaA could be toxic to cells, although a limited increase in the amount of DnaA may be tolerated (52). This encouraged us to test whether a more limited increase in DnaA, which by itself would not be lethal, could bypass growth arrest due to the membrane stress. For this, *Δlon* cells carrying PBAD*dnaA* plasmid were grown without arabinose up to an OD_600_ of 0.1, and the cultures were spread on agar plates containing different amounts of arabinose. The cells collected from the plates were tested for the presence of DnaA by immunoblotting. The data confirmed overproduction of DnaA in the presence of arabinose (Fig. 5F, *left panel*). Increasing the DnaA expression in *Δlon* cells did reduce growth (about fourfold at the highest inducer concentration of 0.1% (Fig. 5G, *left panel*). To test whether this level of overproduction could overcome the membrane stress, the *Δlon* cells carrying the PBAD*dnaA* plasmid were further transformed with the stressor plasmid (carrying *lpp*(C21G)). Coexpression of *lpp*(C21G) and *dnaA* further reduced cell growth (Fig. 4G, *left* vs. *right panels*). Immunoblotting results indicated that in the presence of pLpp(C21G), even when *dnaA* expression was induced, DnaA was proteolyzed (Fig. 5F, *right panel*). The flow cytometry results also indicated a severe loss of DNA upon induction of *lpp*(C21G) under conditions of *dnaA* overexpression (in the presence of 50 µM IPTG and 0.1% arabinose) (Fig. 5H). Together, these results indicate that in the absence of membrane stress, overproduction of DnaA itself could be lethal due to hyper-replication initiation, and upon the membrane stress, the lethality could be from DnaA proteolysis. In the latter case, DnaA was proteolyzed even when overproduced.

### Stress induction in ClpP-deleted cells retains DnaA and does not arrest growth

Our results so far indicate that upon induction of the membrane stress, DnaA is lost without requiring the Lon protease (Fig. 5B). Bacteria can respond to the extracytoplasmic stress via activating, in addition to Rcs (29–31), a two-component system, Cpx (35). The Cpx system controls the activation of the periplasmic DegP protease involved in the clearance of aggregated proteins from the periplasmic space and IM (34). Moreover, the cytoplasmic ClpP protease could also mitigate the stress upon accumulation of mislocalized proteins (35). To test the role of these proteases, we transformed *E. coli* ∆*degP* and ∆*clpP* cells with the stressor plasmid. Cell viability was tested with and without induction of the stressor gene. The ∆*degP* cells expressing the stressor did not grow (Fig. S2). Immunoblotting showed loss of DnaA in such cells. The stress response thus can happen without the DegP protease.

**Fig 6:**
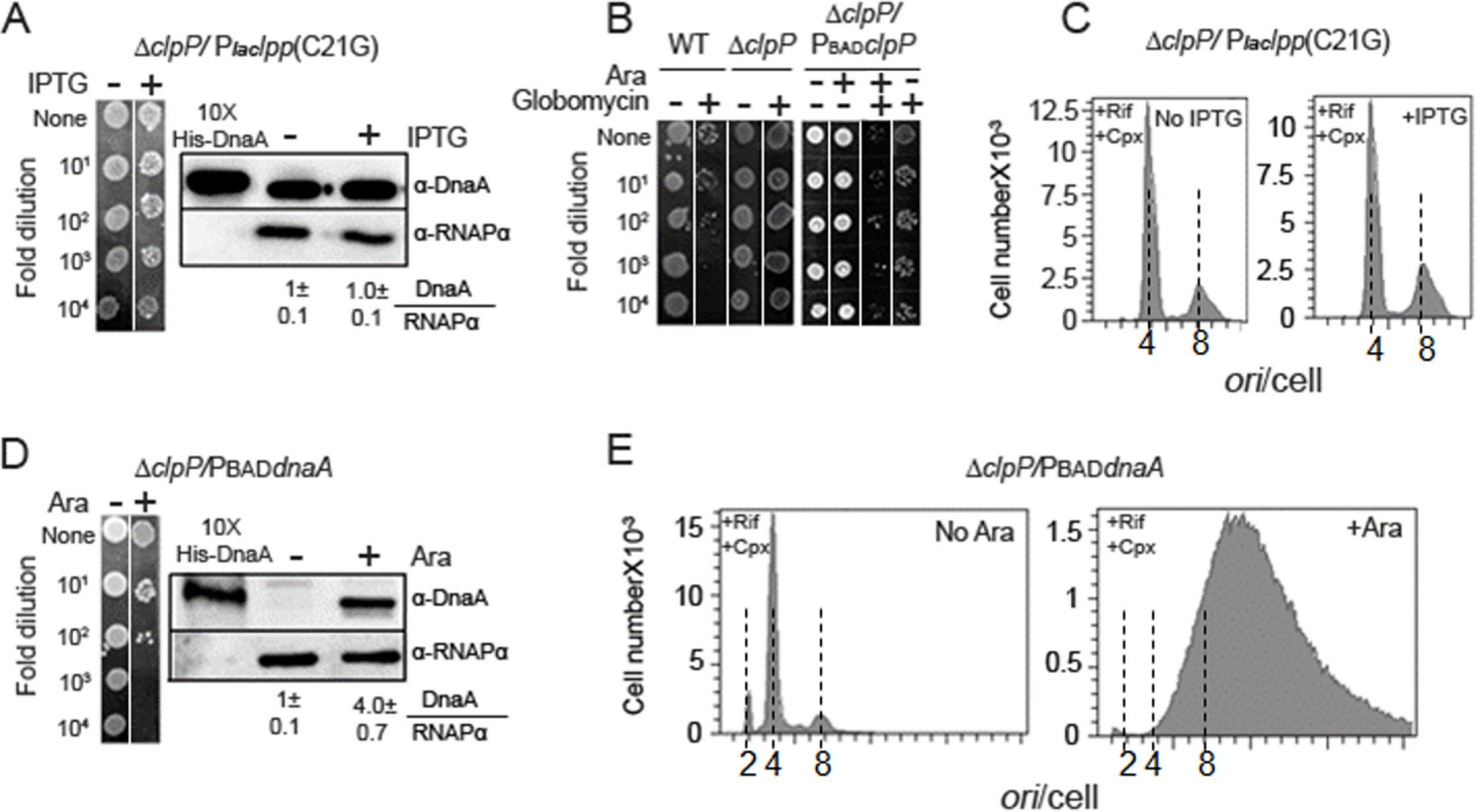
ClpP dependence of DnaA depletion and replication blockage upon membrane stress. (**A**) *E. coli* Δ*clpP* cells transformed with a plasmid carrying the stressor gene P*laclpp*(C21G) were grown without and with IPTG. Cell viability was assayed by spotting (*left panel*) and DnaA content of such cells by immunoblotting (*right panel*). Other details are the same as in Fig. 5A. (**B**) Complementation of ∆*clpP* cells with PBAD*clpP* plasmid. The ∆*clpP* cells resist growth arrest upon exposure to the stressor globomycin, but the complemented cells are not (the last columns of *left* and *right panels*). (**C**) Replication run-out profiles analyzed by flow cytometry of cells as in (**A**). (**D**) Growth (*left panel*) and DnaA content (*right panel*) of Δ*clpP* cells transformed with PBAD*dnaA* plasmid, before and after induction of DnaA by arabinose. Other details are the same as in (**A**). (**E**) Replication run-out profiles before and after induction of DnaA expression of the cells in (**D**).

By contrast, we found that stress induction is not lethal in *∆clpP* cells (Fig. 6A, *left panel*). The result was in line with the immunoblotting data, which showed the presence of DnaA upon stress induction in *∆clpP* cells (Fig. 6A, *right panel*). Results were similar when the stress was induced using globomycin rather than expressing *lpp*(C21G) (Fig. 6B). Complementing ∆*clp* cells with a plasmid with the *clp*^+^ gene returned the growth arrest phenotype upon induction of the stress (Fig. 6B, the last column). Flow cytometric analyses confirmed normal DNA replication in *∆clpP* cells without and with stress induction (Fig. 6C). As would be expected from DnaA stability, the *∆clpP* cells were still susceptible to lethality from *dnaA* overexpression (Fig. 6D) and consequent hyperinitiation (Fig. 6E). In sum, it appears that interfering with pLpp maturation blocks cell growth by proteolyzing DnaA that requires ClpP.

### Overexpression of *dnaA* overcomes the growth arrest in ∆*crp* cells, although not in wild-type cells

Although ClpP appears to be the protease that targets DnaA, how blocking Lpp maturation leads to activation of the protease remains to be understood. Since we ruled out (p)ppGpp as the signaling molecule in the membrane-stress response (Fig. 5A), we considered the role of another common second messenger, cyclic (3′, 5′)-adenosine phosphate (cAMP) (53, 54), in downstream signaling of the membrane stress that culminates in ClpP-mediated DnaA loss.

**Fig 7:**
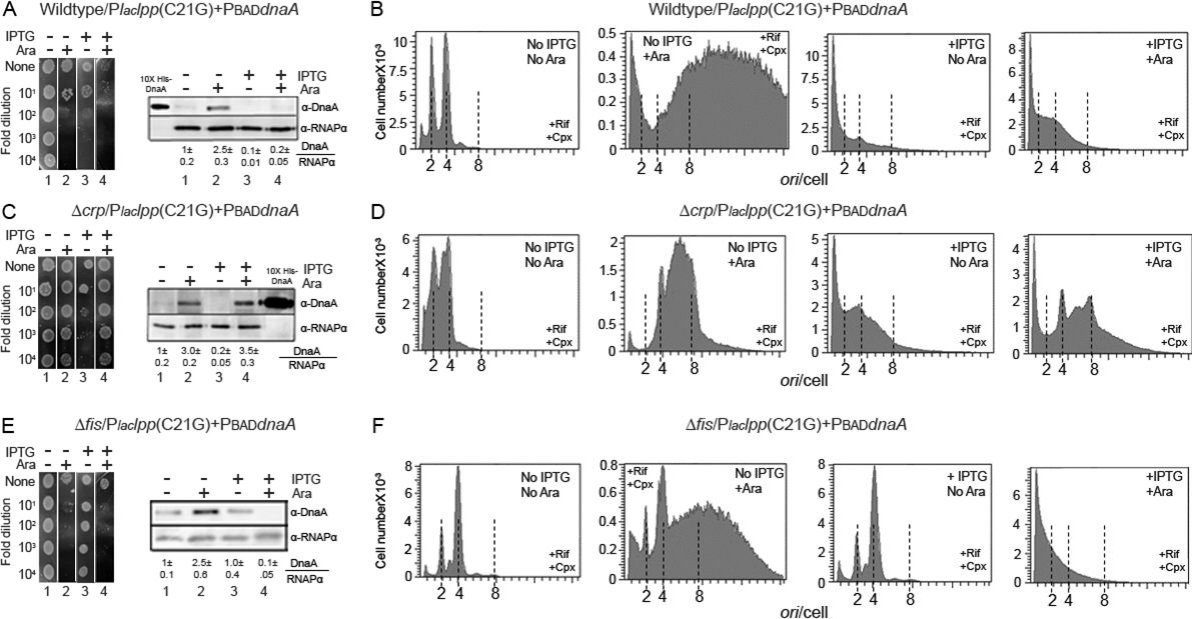
Overcoming membrane stress in Δ*crp* and Δ*fis* cells. *E. coli* wild-type, ∆*crp* and Δ*fis* cells were transformed with a single plasmid containing PBAD*dnaA* or in conjunction with another plasmid containing P*laclpp*(C21G). (**A**) The effect of DnaA overexpression on the growth of wild-type cells in the absence or presence of membrane stress (i.e., ±IPTG) was tested by the spotting assay (*left panel*) and DnaA content of such cells by immunoblotting (*right panel*). (**B**) DNA content of the cells in (**A**) as tested by flow cytometry. (**C, D**) Same as (**A, B**) except that the cells were ∆*crp.* (**E, F**) Same as (**A, B**) except that the cells were ∆*fis*.

We initially tested the role of CRP in the absence of stress induction. The wild-type and *∆crp* cells were transformed with plasmids carrying PBAD*dnaA* and only PBAD (as a negative control). When DnaA synthesis was induced by adding arabinose, only *∆crp* cells showed growth but not the wild-type cells (compare Fig. 7A and C, *left panel lanes 1* vs*. 2*). Immunoblotting indicated similar expression of DnaA in wild-type and *∆crp* cells (Fig. 7A and C, *right panel lanes 1* vs*. 2*). Flow cytometric analysis of cells without the inducer showed roughly similar DNA content (primarily with 2 and 4 chromosomes) in wild-type and *∆crp* cells (Fig. 7B vs. D, no IPTG/no Ara). When DnaA synthesis was induced, there was massive hyperinitiation in wild-type cells but not in ∆*crp* cells (Fig. 7B vs. D, no IPTG/+Ara). These results indicate that the massive hyperinitiation could be responsible for the inviability of wild-type cells, as was seen earlier for *∆lon* cells (Fig. 5E). The hyperinitiation was less in *∆crp* cells, and they were viable. ∆*crp* cells thus provided an opportunity to test whether overexpression of *dnaA* could overcome the growth arrest upon induction of the membrane stress.

The wild-type and *∆crp* cells containing the PBAD*dnaA* plasmid were further transformed with the stressor plasmid, and the transformants were selected on plates without induction of the stressor gene. Induction (by IPTG) inhibited growth and loss of DnaA for both the wild-type and *∆crp* cells (Fig. 7A vs. C, *lane 3*). In agreement, replication was also blocked (Fig. 7B vs. D, +IPTG/No Ara). These results indicate that CRP is not required for the lethality from membrane stress. However, when production of both DnaA and pLpp(C21G) was induced, growth was not arrested in *∆crp* cells, unlike the situation in wild-type cells (Fig. 7A vs. C, *lane 4*). Immunoblotting of wild-type cells showed loss of DnaA (Fig. 7A, *lane 4*), which was also evident by reduced replication events in flow cytometric assays (Fig. 7B, +IPTG/+Ara). In other words, the stress induction is inducing a protease, which is depleting DnaA despite its overproduction. However, in *∆crp* cells, although the induced DnaA level was not altered significantly upon the stress induction (Fig. 7C, *lanes 2* vs. *4*), hyperinitiation was modest (Fig. 7D, +IPTG/+Ara), explaining how cells could be viable. Like in wild type, ∆*crp* cells, when transformed with the P*laccat* plasmid, showed resistance to chloramphenicol when tested without inducing *cat* expression (Fig. S3A). We also verified that the P*laccat* was not inhibited in cells overexpressing DnaA (Fig. S3C), and DnaA overexpression was lethal when ∆*crp* cells were complemented with a P*laccrp* plasmid, as is the case for wild-type cells (Fig. S3B and S3D). These results indicate that overcoming the membrane stress can be achieved by DnaA overproduction, but its activity needs to be toned down to avoid lethal hyperinitiation.

### Stress induction in ∆*fis* cells retains DnaA and does not arrest growth

We formerly found that cells absent the Fis (Factor for inversion stimulation) protein, which negatively regulates replication initiation from *oriC*, are not growth-arrested when they lack PG (*ΔpgsA*) or express the stressor *lpp*(C21G) gene (14; Fig. 7E, *lanes* 1 vs. 3). In line with these results, *∆fis* cells, when treated with globomycin, also did not exhibit growth arrest (Fig. S4). Immunoblotting showed that DnaA is not lost in *∆fis* cells upon induction of the membrane stress with IPTG or upon treatment with globomycin (Fig. 7E, *lanes 1 vs. 3* and Fig. S4). The flow cytometric data also agreed with the inference that upon induction of the membrane stress, *∆fis* cells are not blocked for replication (Fig. 7F, *panels 1* vs *3*). Similarly, increasing DnaA content in *∆fis* cells led to hyperinitiation-mediated growth inhibition as in wild-type cells (Fig. 7E, *lane 2* and Fig. 7F, 2nd *panel*). In addition, the membrane stress in conjunction with DnaA overproduction led to growth arrest due to loss of DnaA and DNA (Fig. 7E, *lane 4* and Fig. 7F, 4th *panel*). These results demonstrate that *∆fis* cells are resistant to membrane stress like the ∆*crp* cells but without requiring help from *dnaA* overexpression.

## DISCUSSION

**Fig 8:**
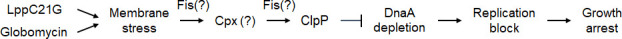
Requirement of ClpP for growth arrest upon membrane stress. How ClpP is activated upon membrane stress is not clear, but a likely candidate is via the Cpx pathway. The requirement of Fis for the growth arrest is more definite, but at what stage of the stress-response pathway is unclear because the details of the pathway itself remain largely unknown, except that Lon, DegP, (p)ppGpp, and CRP are not required.

Stress conditions such as nutrient starvation, fatty acid limitation, and antibiotic treatment accumulate (p)ppGpp molecules (36–38), which cause many regulatory changes, including activation of the Lon protease (39). Lon degrades, among others, DnaA and thus can block new rounds of replication (40, 41, 48, 49). Here, we find that the induction of membrane stress due to interrupted Lpp maturation activates the ClpP protease that degrades DnaA (Fig. 8). This reduces chromosomal DNA drastically, which would suffice to explain cell-growth arrest upon the stress inductions. Here, the stress-response pathway appears novel as it is independent of several stress-response regulators such as Lon, DegP, and the alarmone (p)ppGpp. An independent report indicated that lethality due to the interrupted trafficking of Lpp protein from IM to OM is mitigated in the absence of the two-component system CpxAR (55). Although no report has suggested any relationship between the activation of the CpxAR system and ClpP, our results suggest that the two could be causally related. The details of the pathway remain to be delineated, but Fis protein seems to be a requirement.

In *E. coli* and *Salmonella enterica*, Fis is essential for regulating genes involved in stress-response pathways, particularly those related to defense by antioxidants (56, 57). This function enables cells to effectively counteract oxidative damage. Moreover, Fis plays a pivotal role in activating genes associated with nutrient uptake and metabolism, ensuring optimal resource utilization (58). We previously showed that the Lpp mutant pLpp(C21G), whose accumulation in the IM causes the membrane stress in wild-type cells, is not accumulated in *∆fis* cells, and these cells are not growth-arrested (14). In agreement, we find here that treatment with globomycin, which arrests growth of wild-type cells efficiently but not of *∆fis* cells (Fig. 7E; Fig. S4). This is expected when the interruption of pLpp maturation does not stress cells, as in ∆*fis* cells, inhibiting players in the Lpp maturation pathway, such as LspA, by globomycin, should be inconsequential. Furthermore, we found similar DnaA content and replication initiation frequency in *∆fis* cells without and with membrane stress. The involvement of a global transcription factor like Fis in ClpP-mediated membrane stress indicates that there are likely more genes within the stress-response pathway that are yet to be identified.

We also showed that membrane stress upon defective lipoprotein biogenesis is evident equally in wild-type and ∆*crp* cells, indicating that CRP is not a requirement for the growth arrest. Since the CRP function requires its cofactor cAMP, a well-known nucleotide second messenger, this suggests that the membrane stress is not signaled through cAMP, as is also the case with (p)ppGpp. We note that transcription of the *fis* operon sharply increases during the exponential growth phase, followed by a steep decrease as nutrients become scarce when cells approach the stationary phase (58). It is reported that both CRP and Fis are required to reduce the Fis levels in the cells (59). In other words, the FIS level is expected to stay high in the absence of CRP (59). This scenario is fully consistent with our finding that *∆crp* cells can be growth-arrested (Fig. 7C and D). These cells, however, allowed us to support our central finding that the growth arrest upon the membrane stress is due to DnaA depletion since the arrest is overcome by overexpressing DnaA, which was lethal in wild-type cells but not in ∆*crp* cells. Apparently, the overexpression compensates for the DnaA loss from the membrane stress, allowing replication initiation and cell growth (Fig. 7C and D). In summary, although we have some understanding of the players in the stress-response pathway, much remains to be done to identify particularly the initial players of the pathway.

Our results suggest that globomycin, although known as the direct inhibitor of the LspA enzyme, its bactericidal effect appears to be from DnaA proteolysis. A deeper understanding of the origin of membrane stress and the stress response that results in DnaA degradation is likely to provide further clues to prevent cell growth, considering that DnaA is a universally conserved protein and vital to cell growth. The understanding can be of significant relevance to global health, considering that extended-spectrum β-lactamase-producing bacteria, such as *E. coli, Klebsiella pneumoniae, S. enterica*, and carbapenem-resistant *Enterobacteriaceae*, are prevalent in healthcare settings and becoming drug resistant at an alarming rate (60, 61).

## MATERIALS AND METHODS

We purchased restriction enzymes from New England Biolabs (NEB). The PCR primers used in this study were custom synthesized from Integrated DNA Technologies. We performed polymerase chain reactions using Q5 high-fidelity DNA polymerase (NEB). Chemical ingredients to make buffers and growth media were purchased from Sigma or VWR.

### Bacterial strains and plasmids

Bacterial strains and plasmids used in this study are mentioned in Table S2.

### Growth assay

Single transformants obtained by transforming wild-type (*pgsA^+^*) cells with plasmid DNA carrying *lpp*(WT), *lpp*(C21G), or *lpp*(C21G/∆K) genes placed under the inducible P*lac* promoter were inoculated in M9 + Glu medium supplemented with ampicillin (100 µg/mL). The cultures grown overnight under non-inducing conditions were diluted in M9 +Glu medium to an OD_600_ of 0.005. The cultures were allowed to grow for an additional 45 minutes before dividing into two aliquots, with one containing isopropyl-β-D-galactopyranoside (IPTG) (50 µM) or globomycin (10 µg/mL) antibiotic. The samples were collected at the indicated time intervals to monitor the growth of cells carrying normal and mutant *lpp* genes.

### Immunoblotting

At the last time point of growth assays, the cells were pelleted by centrifugation at 16,000 *× g* for 10 min at 4°C. Cell pellets were resuspended in 500 µL of PBS, and lysates were prepared by sonication for 5 minutes (in cycles of 10″ off and 10″ on). Following the determination of protein concentrations by the Bradford assay, protein aliquots were stored at −80°C until use. Lysates containing 5 µg of protein mixed with 1× SDS sample buffer were boiled, and the proteins were resolved on 12% SDS-PAGE. Proteins transferred to PVDF membranes were immunoblotted with polyclonal α-DnaA antiserum and treated with stabilized peroxidase-conjugated secondary antibody (Thermo Fisher Scientific). Blots were reprobed with monoclonal α-RNAP (alpha subunit), whose amounts served as loading controls. Immunoblots were visualized using an AI600 Imager. The DnaA/RNAPα ratios are normalized with respect to the *lpp*(WT) value of 1. The data presented are from at least three biological replicates.

### Spotting assay

The transformants were selected on M9-Agar supplemented with appropriate antibiotics but without IPTG. Single transformants were grown at 37°C in M9 + Glu media to an exponential phase (OD_600_ of 0.10). To perform the spotting assay, serial dilutions were prepared, and 2.5 µL from each sample was placed on the M9-Agar + Glu media with or without globomycin. Plates were allowed to grow at 37°C for 16–24 hours. The results of spotting assays are presented in a single panel, which may contain data from different plates (e.g., ±IPTG and ±globomycin). Growth should be compared vertically within a column rather than between columns.

### Plating assay

*E. coli* WT (*pgsA^+^*) cells transformed with single or the two-plasmid DNAs were selected on the M9-Agar + Glu containing plates supplemented with the appropriate antibiotics (ampicillin: 100 µg/mL, tetracycline: 12.5 µg/mL, kanamycin: 50 µg/mL). The viability of bacterial cells expressing DnaA(WT) alone or in conjunction with mutant pLpp(C21G) was tested. For this, single transformants containing either a single or two plasmids were inoculated in M9 + Glu media to an exponential phase (OD_600_ of 0.10). (Note that cells expressing pLpp(C21G) grow up to OD_600_ of 0.10 (Fig. 2A)). Dilutions were prepared and plated to obtain 300–500 colonies on the M9-Agar plates containing the appropriate antibiotics, with or without IPTG, arabinose, or both.

### Flow cytometry

Flow cytometry was performed as mentioned earlier (16). Briefly, overnight-grown cells were diluted to an OD_600_ of ~0.005 in LB media supplemented with appropriate antibiotics. The cultures were divided into two equal volumes, to one of which IPTG (50 µM) was added to induce expression of different *lpp* alleles. When desired, cells were further grown to an OD_600_ of 0.15 and treated with rifampicin and cephalexin to inhibit new rounds of initiation and cell division, respectively. Cells were grown for an additional 2.5 hours to allow completion of ongoing rounds of replication and were subsequently analyzed for replication parameters. In all experiments, 100,000 cells were counted. In flow cytometry profiles, cells with 2 and 4 origins could be recognized in most control conditions (without IPTG, arabinose, or globomycin treatment). The distance between 2 and 4 origin cells was used as a guide to mark *ori*/cell in experimental cells, especially when clear peaks were not apparent. The major peak of the profile was assumed to have four chromosomes in ambiguous situations.

### QRT-PCR

Cells grown in LB or M9 media were lysed at 4°C using disruptor beads and vortexing. Total RNA was extracted, and samples were treated with RNase-free DNase to eliminate possible genomic DNA contamination. RNA concentration and integrity were assessed before performing cDNA synthesis. Each real-time PCR was limited to 40 cycles, and melt-curve analysis was performed to ensure fidelity of the amplicon. Data were normalized to the ∆Cq value of a commonly used reference gene, *rrsA* (16S rRNA gene)*,* and the fold changes in the expression of the target genes were estimated.

## SUPPLEMENTAL MATERIAL

Supplemental tables and figures jb.00151-25-s0001.pdf Tables S1 and S2, and Fig. S1 to S4.

ASM does not own the copyrights to Supplemental Material that may be linked to, or accessed through, an article. The authors have granted ASM a non-exclusive, world-wide license to publish the Supplemental Material files. Please contact the corresponding author directly for reuse.
