# Supplementary material for: A novel membrane stress response that blocks chromosomal replication by targeting the DnaA initiator via the ClpP protease
Source: J Bacteriol. 2025 Jun 24;207(7):e00151-25. doi: 10.1128/jb.00151-25 (PMC12288453; doi:10.1128/jb.00151-25)
Supplement: Supplemental tables and figures — Tables S1 and S2, and Fig. S1 to S4. [file jb.00151-25-s0001.pdf]

Supplemental Information

A novel membrane stress-response that blocks chromosomal replication by targeting the DnaA initiator via the ClpP protease

Alabi Gbolahan[1#], Tong Li[1], Rishit Saxena[1], Karen Wolcott[2], Aamna Sohail[1], Ishika Ahmed[1], Dhruba K. Chattoraj[2], Elliott Crooke[1, 3], and Rahul Saxena[1#*]

[1]Department of Biochemistry and Molecular & Cellular Biology, Georgetown University Medical Center, Washington, DC 20007, USA

[2]Basic Research Laboratory, Center for Cancer Research, National Cancer Institute, National Institutes of Health, Bethesda, Maryland, USA

[3]Lombardi Comprehensive Cancer Center, Georgetown University Medical Center, Washington, DC 20007, USA

Running title: Membrane-stress associated replication block.

[#] Authors contributed equally.

*To whom correspondence should be addressed: Tel: +1-202-687-1642; Fax: +1-202-687-7186; E. mail: rs426@georgetown.edu

Table S1: Growth parameters for *E. coli* with membrane-stress

	***E. coli pgsA*$^{+}$*dnaA*$^{+}$**							
	Generation time (min)				Growth rate/hr			
	P_{lac}EV	P_{lac}*lpp* (WT)	P_{lac}*lpp* (C21G)	P_{lac}*lpp* (C21GΔK)	P_{lac}EV	P_{lac}*lpp* (WT)	P_{lac}*lpp* (C21G)	P_{lac}*lpp* (C21GΔK)
	46.4 ± 8.5	49.7 ± 8.7	83.7 ± 1.9	51.7 ± 7.1	0.91 ± 0.16	0.85 ± 0.14	0.50 ± 0.08	0.82 ± 0.11
	***E. coli pgsA*$^{+}$*dnaA*$^{+}$**							
	No Globomycin		Globomycin		No Globomycin	Globomycin		
	30.2 ± 0.1		50.4 ± 4.8		1.38 ± 0.01	0.81 ± 0.08		
	E. coli pgsA*$^{+}$Δ*dnaA							
	46.0 ± 1.5	42.5 ± 2.8	46.1 ± 3.9	43.8 ± 2.5	0.91 ± 0.03	0.98 ± 0.07	0.91 ± 0.08	0.95 ± 0.05

Table S2: *E. coli* strains and plasmids used in this study

Strain designation*	Genotype
BW25113= wildtype	F-, *Δ(araD-araB)567*, *ΔlacZ4787*, rrnB-3, λ^-, *rph-1*, *Δ(rhaD-rhaB)568, hsdR514*
JW1667-5	BW25113 ***Δlpp-752::kan***
JW2755-3	BW25113 ***ΔrelA782::kan***
JW0429-1	BW25113 ***Δlon-725::kan***
JW0427-1	BW25113 ***ΔclpP723::kan***
JW5702-4	BW25113 ***Δcrp-765::kan***
JW5702-4	**BW25113Δ*fis*-765::kan**

Plasmids	Relevant features	Source
P*laclpp*(C21G)	pBR322: *lacI*, *lpp*^{P}-*lac*^{PO} -*lppCys*^{21}-*Gly:*Amp^R	5, 14
P*laclpp*(WT)	pBR322: *lacI*, *lpp*^{P}-*lac*^{PO} -*lpp:* Amp^R	14
P*laclpp*(C21GΔK)	pBR322: *lacI*, *lpp*^{P}-*lac*^{PO} -*lpp:* Amp^R	This study
P*lac-cat*	pBR322: *lacI*, *lpp*^{P}-*lac*^{PO} -*cat:* Amp^R, Cm^R	This study
P*lac-crp*	pBR322: *lacI*, *lpp*^{P}-*lac*^{PO} -*crp:* Amp^R	This study
pSC(*dnaA*)	p15A: *araC*, P_{BAD-} *dnaA:* Tet^R	14
P_{BAD}*clpP*	pBR322: *araC,* P_{BAD-}*clpP*: Amp^R	1[#]

***All strains are from KEIO collection**

Reference:

1[#]. Camberg JL, Hoskins JR, Wickner S. The interplay of ClpXP with the cell division machinery in Escherichia coli. J Bacteriol. 2011 193(8):1911-1918.

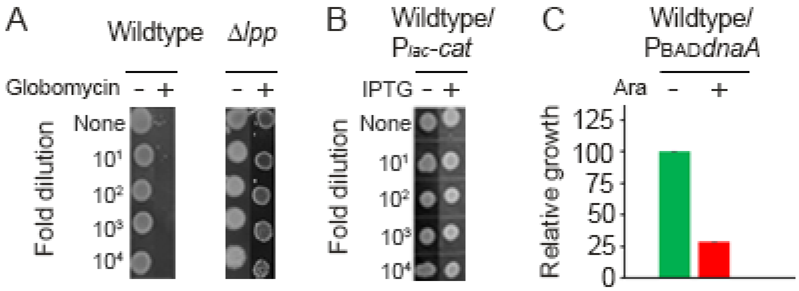

Fig. S1. (*A*) Cell viability of wildtype (*pgsA*$^{+}$ *lpp*$^{+}$) *vs. pgsA*$^{+}$Δ*lpp* cells in the absence or presence of globomycin. *(B)* Growth of wildtype cells expressing *cat* gene plated on the LB-Agar plates supplemented with chloramphenicol in the absence and presence of IPTG inducer. (*C*) Growth of wildtype cells carrying PBAD*dnaA* grown without or with arabinose.

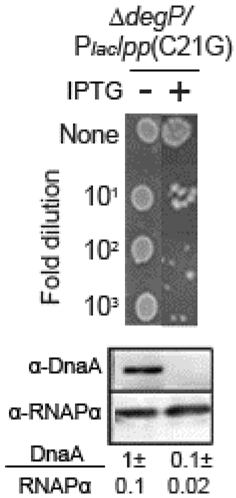

Fig. S2. Growth of *E. coli* Δ*degP* cells transformed with plasmid DNA carrying *lpp*(C21G) placed under P*lac* promoter. The cells were grown without and with IPTG, and their viability was assayed by spotting (*top panel*) and their DnaA content by immunoblotting (bottom *panel*). The α-subunit of RNA polymerase (RNAPα) was used as the loading control. In all the spotting assays, growth should be compared vertically (within a column) rather than between columns, as they are from different plates (e.g., -/+ IPTG).

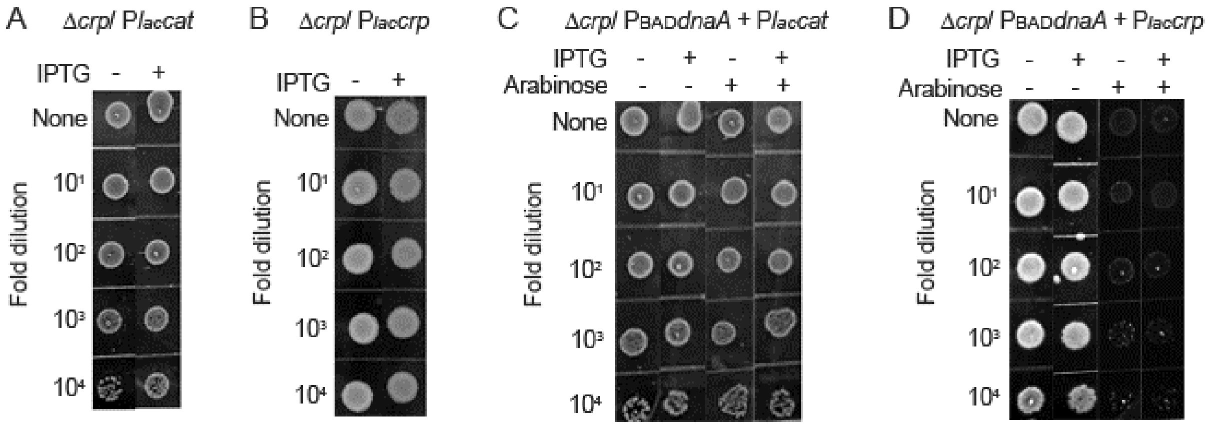

Fig. S3. Uninduced level of (leaky) activity of the P*lac* promoter tested in *E. coli* Δ*crp* cells. The cells were transformed with plasmids containing P*laccat* or P*laccrp*, either alone or in conjunction with a second plasmid containing PBAD*dnaA*. *(A-B)* Growth of Δ*crp* cells containing plasmid P*laccat* (A) on LB-Agar supplemented with chloramphenicol (32.5 µg/mL) or P*laccrp* (B) on LB-Agar containing ampicillin (100 µg/mL) in the absence or presence of inducer (i.e., ±IPTG). *(C-D)* Growth of Δ*crp/*PBAD*dnaA* cells transformed with plasmid containing P*laccat* on LB-Agar supplemented with chloramphenicol and tetracycline (12.5 µg/mL) (C) or transformed with plasmid containing P*laccrp* on LB-Agar supplemented with ampicillin and tetracycline (D). Additionally, the plates contained IPTG and/or arabinose as indicated.

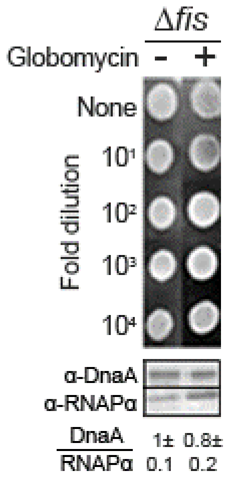

Fig. S4. Insensitivity of Δ*fis* cells to membrane-stress inducing drug globomycin. (*Top*) Spotting assay to test viability of Δ*fis* cells when exposed or not to globomycin. (*Bottom*) Immunoblotting of DnaA and RNAPα from such cells.
